# Substance use among people with severe mental illness during the COVID-19 pandemic and earthquakes: the role of community-based treatment

**DOI:** 10.1192/j.eurpsy.2023.1248

**Published:** 2023-07-19

**Authors:** S. Levaj, S. Medved, J. Gerlach, L. Shields-Zeeman, F. Bolinski, Z. Bradaš, Z. Madžarac, I. Rojnić Palavra, M. Rojnić Kuzman

**Affiliations:** 1 Department of Psychiatry and Psychological Medicine, University Hospital Centre Zagreb; 2 University Psychiatric Hospital Sveti Ivan, Zagreb, Croatia; 3 Netherlands Institute of Mental Health and Addiction (Trimbos Institute), Utrecht, Netherlands; 4 School of Medicine, University of Zagreb, Zagreb, Croatia

## Abstract

**Introduction:**

The COVID-19 pandemic has disrupted mental healthcare delivery in many countries. The restricted access to psychiatric services and double disasters (pandemic and earthquakes) coincided in Croatia, potentially placing people with severe mental illness (SMI) in a very vulnerable position.

**Objectives:**

The aim of this study was to examine the changes in substance use in people with SMI in the first and the second COVID-19 wave and co-occurring earthquakes. The secondary aim was to explore whether the type of treatment (community mental health teams (CMHT) vs. treatment as usual) influenced those changes.

**Methods:**

This study was nested within the RECOVER-E project (LaRge-scalE implementation of COmmunity based mental health care for people with seVere and Enduring mental ill health in EuRopE, Horizon 2020 research and innovation programme, grant agreement No 779362). The study involved 90 participants with SMI assessed at two time points: in May/June 2020 (during the first COVID-19 wave and after Zagreb earthquake) and in December 2020/January 2021 (during the second COVID-19 wave and after Petrinja earthquake). The changes in the use of psychoactive substances (alcohol, cannabis, other drugs, sedatives) were assessed using self-reported survey.

**Results:**

The increase in tobacco smoking behavior and the use of sedatives was observed in both COVID-19 waves in people with SMI. No increase was reported in cannabis and other drugs use, while less than 5% of participants reported increase in alcohol consumption. Not receiving CMHT service predicted the increase in sedative use.

**Conclusions:**

Ensuring accessible mental health care provided by CMHT is recommended for counteracting the negative effect of external stressors (such as pandemic and co-occurring earthquakes) on the increased substance use among people with SMI.

**Disclosure of Interest:**

S. Levaj Grant / Research support from: Project RECOVER-E - European Union’s Horizon 2020 research and innovation programme; Grant Agreement No 779362., S. Medved Grant / Research support from: Project RECOVER-E - European Union’s Horizon 2020 research and innovation programme; Grant Agreement No 779362., J. Gerlach: None Declared, L. Shields-Zeeman Grant / Research support from: Project RECOVER-E - European Union’s Horizon 2020 research and innovation programme; Grant Agreement No 779362., F. Bolinski Grant / Research support from: Project RECOVER-E - European Union’s Horizon 2020 research and innovation programme; Grant Agreement No 779362., Z. Bradaš Grant / Research support from: Project RECOVER-E - European Union’s Horizon 2020 research and innovation programme; Grant Agreement No 779362., Z. Madžarac Grant / Research support from: Project RECOVER-E - European Union’s Horizon 2020 research and innovation programme; Grant Agreement No 779362., I. Rojnić Palavra: None Declared, M. Rojnić Kuzman Grant / Research support from: Project RECOVER-E - European Union’s Horizon 2020 research and innovation programme; Grant Agreement No 779362.

